# Quality of Substance Use History Taking and Drug Screening in Adult Psychiatric Inpatients With Co-Occurring Mental Illness

**DOI:** 10.1192/bjo.2026.11684

**Published:** 2026-06-30

**Authors:** Shreyan Kar, Salma Ghafoor, Suneela Jabeen

**Affiliations:** Black Country Healthcare NHS Foundation Trust, Dudley, United Kingdom

## Abstract

**Aims:**

Comorbid substance misuse is common in patients with mental illnesses, which are associated with challenges in diagnosis, management, poor outcomes, and increased risks. National Institute for Health and Care Excellence guidance recommends detailed history taking, biological testing, and early management. This audit aimed to assess the quality of substance use history taking, urine drug screening, and documentation of management plans for adult psychiatric inpatients.

**Methods:**

Clinical audits were completed across four adult psychiatric hospitals in the Black Country Healthcare NHS Foundation Trust in April 2025 and January 2026. Patients were randomly selected and stratified by site and sex. Information about substance use, urine drug screening, type of substances, and management of substance use was collected along with relevant clinical history. The first 48 hours of admission documentation were reviewed. Data were collected using a predesigned questionnaire from the electronic patient records. Following the initial audit, efforts were taken to increase awareness among clinicians and provide recommendations.

**Results:**

There were 40 patients in each of the audits (age range: 19-73 years). Diagnoses were comparable: psychotic disorders (47.5% and 47.5%), mood disorders (40% and 30%), and personality disorders (12.5% and 22.5%). Most patients had a substance use history, 82.5% and 72.5% in the first and second audit, respectively. Urine drug screening within 48 hours of admission increased between cycles (42.5% vs. 50%); with more than half having positive results (58.8% vs. 55%).Commonly used substances were benzodiazepines (45.9%) and tetrahydrocannabinol (18.9%), along with opiates, ketamine, cocaine, and amphetamines. The reasons for lack of screening in the first and second audits were patient refusals (39.5%); there were delayed testing in 9.3%, and no documented reasons in 57.5% of cases. Drug use screening questionnaire usage remained low, but increased from 0% to 5%. There were changes in the documentation of related history such as accommodation (50% vs. 85%, p<0.001), personal history (57.5% vs. 65%), past medical history (92.5% vs. 87.5%), social history (82.5% in each cycles), and forensic history (60% and 75%) in first and second audit respectively. Discussion of substance use management was documented more frequently (35% to 75%, p<0.001).

**Conclusion:**

More than half of the adult inpatients who underwent drug screening had a positive result. Substance use-related management discussion became significantly more frequent in the second audit. Patient refusals for drug screening remain a major concern. There is a continued need to monitor drug screening and management in adult psychiatry wards.

